# Clinical outcome comparison of polymethylmethacrylate bone cement with and without mineralized collagen modification for osteoporotic vertebral compression fractures

**DOI:** 10.1097/MD.0000000000012204

**Published:** 2018-09-14

**Authors:** Xi Wang, Jian-Ming Kou, Yang Yue, Xi-Sheng Weng, Zhi-Ye Qiu, Xi-Feng Zhang

**Affiliations:** a Department of Orthopedics, Lianyungang Second People's Hospital, Lianyungang Jiangsu; b Department of Orthopedics, Peking Union Medical College Hospital; c School of Materials Science and Engineering, Tsinghua University; d Department of Orthopedics, Chinese PLA General Hospital, Beijing, China.

**Keywords:** mineralized collagen, osteoporosis, PMMA bone cement, vertebral compression fracture

## Abstract

A retrospective study of consecutive patients.

The purpose of this study was to compare the clinical effect of biomimetic mineralized collagen (MC) modified polymethylmethacrylate (PMMA) bone cement and traditional PMMA bone cement for the treatment of osteoporotic vertebral compression fractures (OVCF).

New fracture on adjacent level is the major postoperative complication of percutaneous vertebroplasty (PVP). The clinical incidence was 12.4% to 27.7%. The increased stiffness of the treated vertebral body caused by filling bone cement is considered as one of the main reasons.

A total of 30 patients treated with traditional PMMA bone cement from June 2013 to March 2016 were selected as the traditional group, while 50 patients treated with MC modified PMMA bone cement from July 2014 to March 2016 were selected as the modified group. The 2 groups were compared by injection time of the bone cements, postoperative pain relief effects, vertebral height restoration, CT value changes of the treated vertebral bodies, and postoperative complications in the clinical observations.

The surgeries were successfully completed in both groups. In the treatment of OVCF, the MC modified bone cement was able to achieve the same pain relief and vertebral height restoration effects compared to traditional bone cement during the follow-ups, although the injection time of the cement was prolonged in the operation. MC modified bone cement significantly reduced the incidence of postoperative adjacent vertebral fracture from 13.3% to 2%, and significantly increased bone density of the treated vertebral bodies.

The MC modified PMMA bone cement showed good clinical outcomes and better mechanical properties than the traditional bone cements.

## 1 Introduction

With the aging of the population, the annual incidence of osteoporotic vertebral compression fractures (OVCF) in China has been increasing. The minimally invasive percutaneous vertebroplasty (PVP) provides obvious relief of pain and allows for early postoperative ambulation. Therefore, it has been widely preferred as the treatment for elderly patients with OVCF. However, high incidence of postoperative adjacent vertebral fractures, which was 12.4% to 27.7% according to previously published literatures, has severely undermined patients’ quality of life.^[1,2]^ In most cases, additional PVP surgeries are required at these fractured segments, inflicting pain and financial burden on the patients.

Many studies have been conducted around the world in order to modify and enhance the biomechanics and biocompatibility of polymethylmethacrylate (PMMA) bone cement. Ingredients such as hydroxyapatite, chitosan, or sodium hyaluronate have been added to bone cement, and other monomers or liquids to MMA monomers. However, an ideal technical method that meets clinical demands still remains to be achieved.^[3–8]^ The disadvantages of the present methods have been revealed. For example, the addition of hydroxyapatite to PMMA bone cement increases elastic modulus of the solidified body,^[4]^ while other monomers added in MMA monomers can decrease strength.^[8]^

Based on previous studies on biomimetic mineralized collagen (MC) bone repair materials and their clinical applications, biomechanics and biocompatibility of current clinically available PMMA bone cements could be improved by adding an appropriate proportion of the MC.^[9]^ The biomimetic MC consists of orderly arranged type I collagen and nano-hydroxyapatite. With its components and microscopic structure consistent with natural bone, MC features good osteogenic activity, and has been clinically used for bone defect repair in a wide range.^[10–14]^ As demonstrated in previous studies, compound modification by using MC can significantly reduce elastic modulus of PMMA bone cement without influencing the strength, as well as improving compatibility with adjacent bone tissue to form osseointegration.^[9,15,16]^ Our department has been using MC modified PMMA to treat OVCF since June 2013 and has compared treatment outcomes with traditional PMMA. Clinical observations were followed-up and outcomes were analyzed.

## 2 Methods

### 2.1 Study design

**Figure 1:**
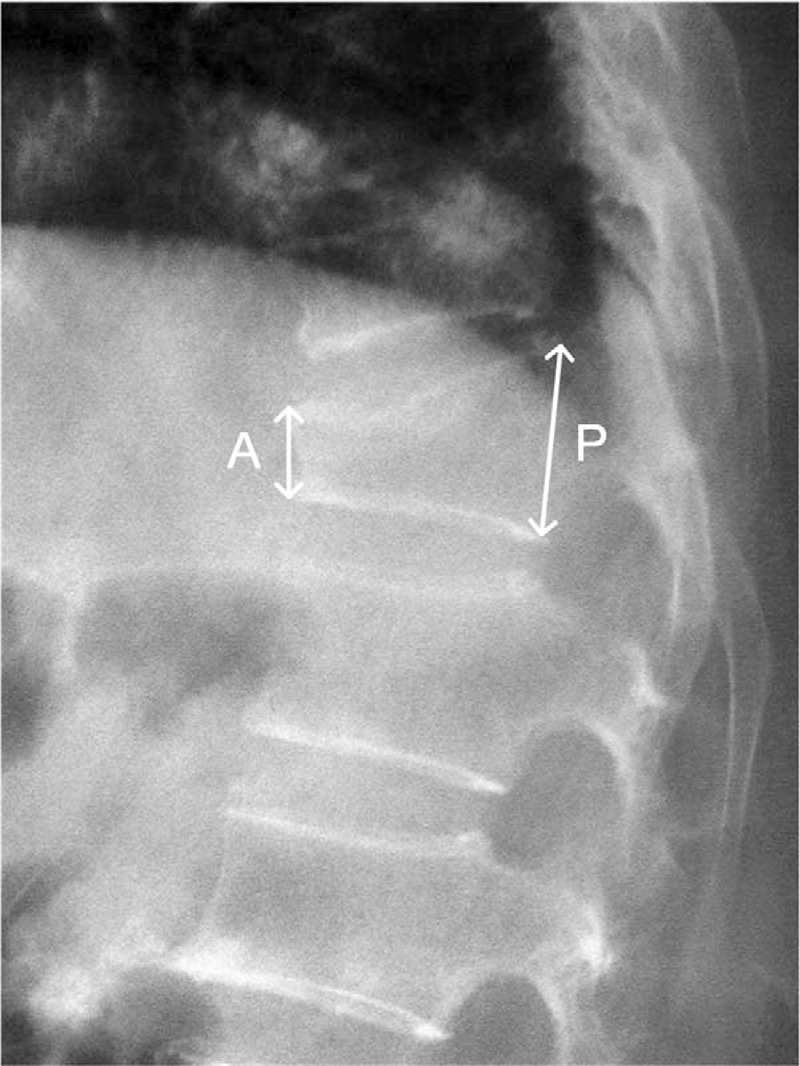
Measurement of anterior wall (A) and the posterior wall (P). The anterior–posterior ratio was calculated as A divided by P.

We evaluated 30 patients with single OVCF treated with traditional PMMA in our department between June 2013 and March 2016 (traditional group) and 50 patients with single OVCF treated with MC modified PMMA in our department between July 2014 and March 2016 (modified group). There were 8 males and 22 females in the traditional group. Age ranged from 56 to 88 (average, 72.80). There were 6 males and 44 females in the modified group. Age ranged from 57 to 90 (average, 76.16). General data comparisons between the 2 groups are listed in Table 1. There was no significant difference for age, gender, and bone mineral density between the 2 groups. The patients all recalled a history of trauma and vertebral compression fracture within 3 weeks. Bone densitometry was used before surgery to confirm osteoporosis. Vertebral x-ray, MRI, and CT were performed to exclude occupying lesions of vertebral bodies, intervertebral disc diseases, or spinal stenosis, which could also cause pain due to dural sac or nerve root compression. CT study was used to confirm posterior wall intactness of fractured vertebral body, unilateral or bilateral pedicle intactness of fractured vertebra, and that no burst occurred in the lower half and inferior end plate of vertebral body. PVP was performed when the ratio of anterior wall to posterior wall was 40% to 80% (Fig. 1). This clinical observation was approved by Ethics Committee of Lianyungang Second People's Hospital.

**Table 1:**
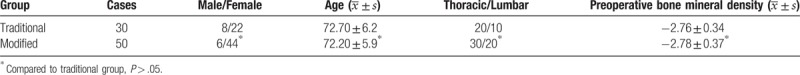
General data comparison between the 2 groups.

### 2.2 Surgical techniques

The patient was laid at a prone position with stomach hanging. ECG and vital signs were monitored throughout the course of surgery. Pedicle(s) of fractured vertebra was located using C-arm x-ray machine and mark the location on the body surface. Disinfection and draping were then performed.

Traditional group: after local infiltration anesthesia, x-ray was used to guide puncturing through the pedicle and establish the working channel. Percutaneous puncturing was performed from the superior lateral direction of the pedicle of fractured vertebra in the AP fluoroscopy (10 o’clock direction in the left, 2 o’clock direction in the right), forming an angle about 13°∼17° (determined based on the anterio-oblique fluoroscopy during surgery) with the sagittal plane. Punctured the needle through the pedicle and to the middle of the vertebral body. Threaded the probe through the working channel to one-third of the vertebral body, then inserted the tapping through the working channel and tapped to one-third anterior of the vertebral body, with ensuring that the tapping reached the midline or slightly further under AP fluoroscopy. After appropriate puncture site was confirmed, removed the tapping. At this time, the assistant mixed bone cement powder and liquid according to the instruction until it reached a drawing stage. The bone cement was injected and the injectable time was recorded, with the whole course of injection under the monitor of the C-arm x-ray machine. Paused injection when the bone cement infiltrated along trabeculae to the margin of the vertebral body and formed a spiculated pattern to the bone cortex. After a few seconds, continued to inject the bone cement and paused when the above-mentioned imaging findings appeared again. Repeated the procedure until the desired anterior vertebral height was achieved. After the injection at the anterior portion of the vertebral body was completed, the injection was performed at the middle portion. The injection was stopped when the bone cement was fully diffused and approached the posterior portion. Recorded the time when bone cement cannot be injected and the bone cement volume injected into the fractured vertebral body.

Modified group: after local infiltration anesthesia, puncture the fractured vertebral body as in the traditional group. The powder and the liquid of the bone cement were blended to form a mixture with low viscosity, and then a certain amount of MC bone graft particles (produced by Beijing Allgens Medical Science and Technology Co., Ltd., China, certificated by US Food and Drug Administration and China Food and Drug Administration) was added. The mixture continued to be blended until it reached a drawing stage. The MC modified bone cement was injected as in the traditional group. The injectable time and the bone cement volume injected into the fractured vertebral body after the modification were recorded. The volume of the added MC bone graft particles was about 15wt% of the powder of the bone cement, since such portion could minimized compressive modulus of the bone cement without decrease its strength and influence the injectability according to our previous studies.^[9]^

During the injection, patient's ECG was closely monitored and patient's overall condition was constantly asked, especially whether numbness or pain in lower extremities or intense back pain was presented to predict and prevent bone cement leakage. After bone cement fully solidified, the needle was removed. After watching for 10 minutes, the operation was completed if sensation and movement were normal and vital signs stable. The patient was laid supinely for 6 hours, then sit up 12 hours after surgery. Early ambulation was encouraged 24 hours after surgery. Multi-modality therapy was administered after the surgery, including calcium supplement and anti-osteoporotic therapies.

### 2.3 Indicators

Record the injectable time in the 2 groups; bone cement volume injected; pain relief assessment based on the visual analog scale (VAS) before surgery, 2 days after surgery and in each postoperative follow-up; vertebral height of the anterior margin and in the middle before surgery and in the follow-ups. The cross section at pedicle level of the fractured vertebra was divided into 9 regions after trisecting longitudinal and transverse axes. Select in each region 1 equally sized circle, measure their corresponding CT values, and calculate their mean CT values before surgery and in each follow-up. Calculate the incidence of postoperative complications.

The clinical diagnostic criterion for postoperative adjacent vertebral fracture was definite vertebral height decrease on plain radiograph and increased signal intensity on MRI due to bone marrow edema inside the vertebral body.^[24]^

### 2.4 Statistical analysis

Statistics were in the form of 
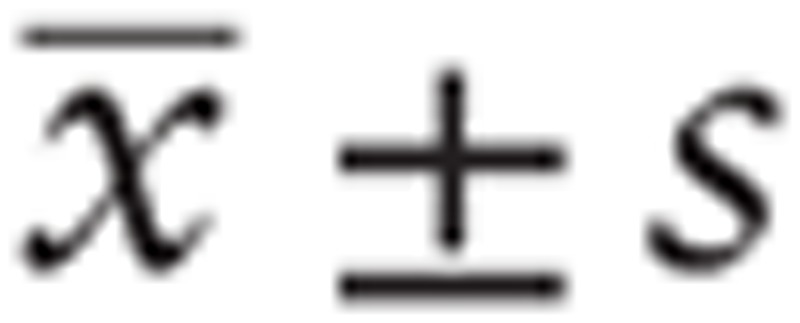
. Statistical analysis was performed with the use of SPSS software, version 16.0 (SPSS). Paired *t* tests were used for the comparison before surgery and in the follow-ups. *χ*^2^ tests were applied to compare the incidence of postoperative complications. *P* < .05 was considered to indicate statistical significance.

## 3 Results

All 80 patients were followed after surgery for 6∼12 months (average, 10.8 months). Comparison of indicators before and after surgery revealed that the modified group had longer injectable time than that of the traditional group, and the difference was statistically significant (*P* < .05); the modified group had noticeably lower incidence of postoperative adjacent vertebral fracture, and the difference was statistically significant (*P* < .05); the modified group had lower incidence of bone cement leakage and larger bone cement injection volume, but the difference was not statistically significant (*P* > .05). No bone cement dislodgment from the posterior margin into the neural canal or clinical presentations caused by bone cement leakage occurred in either group (Table 2).

**Table 2:**
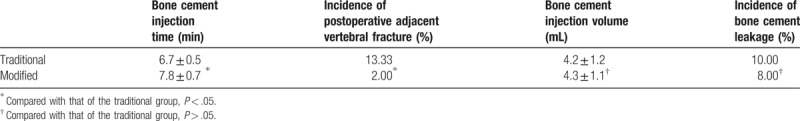
Indicators (
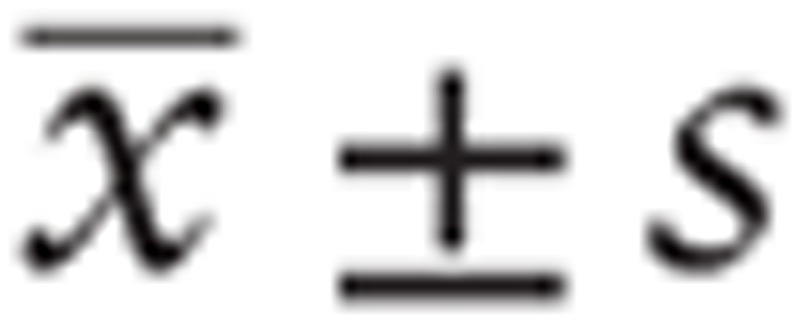
).

**Figure 2:**
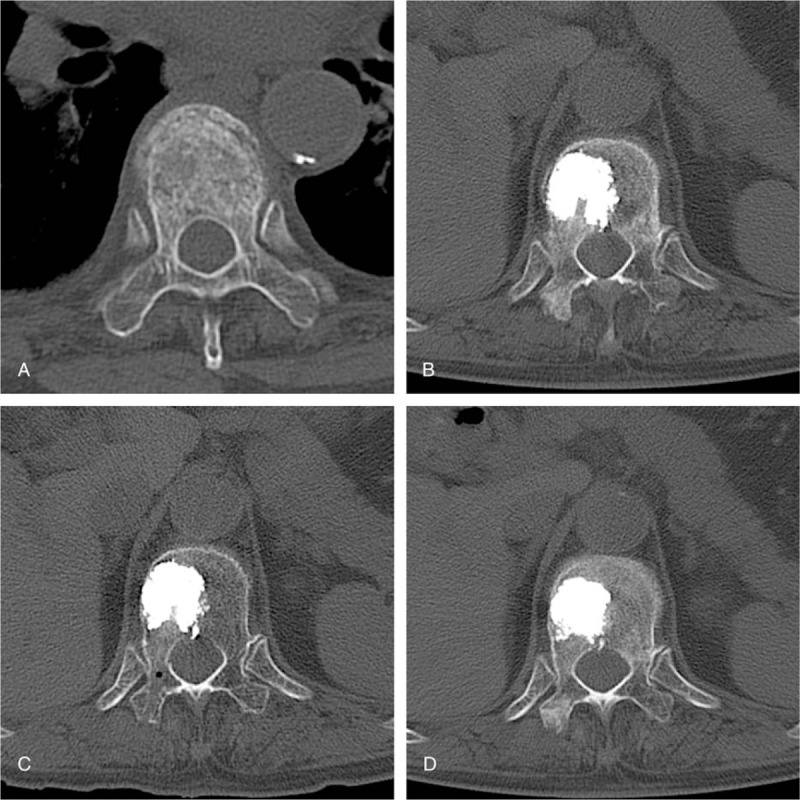
Preoperative examination and follow-up CT films of a typical case in the modified group ([A] preoperative examination, [B] 1 month after the surgery, [C] 6 months after the surgery, 1 year after the surgery).

VAS scores improved at 2 days, 1 month, 3 months, 6 months and 1 year after surgery in both groups compared with those before surgery. The difference was statistically significant (*P* < .05) but was not between groups in each follow-up (*P* > .05) (Table 3). In both groups, the height of the fractured vertebral body showed obvious improvement after surgery than that before, and the difference was statistically significant (*P* < .05); there was loss of height in the fractured vertebra with time, and the loss was more prominent in the traditional group. The difference was statistically significant (*P* < .05) (Table 4). In the modified group, the intra-vertebral CT value in each follow-up increased compared with that before surgery. The difference was statistically significant (*P* < .05) (see Fig. 2). In the traditional group, the intra-vertebral CT value in each follow-up increased compared with that before surgery. The difference was not statistically significant (*P* > .05). The intra-vertebral CT value of the modified group in each follow-up is noticeably higher than that of the traditional group. The difference was statistically significant (*P* < .05) (Table 5).

**Table 3:**
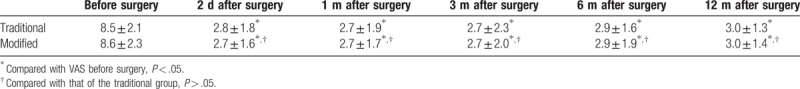
Changes in VAS scores before and after surgery (
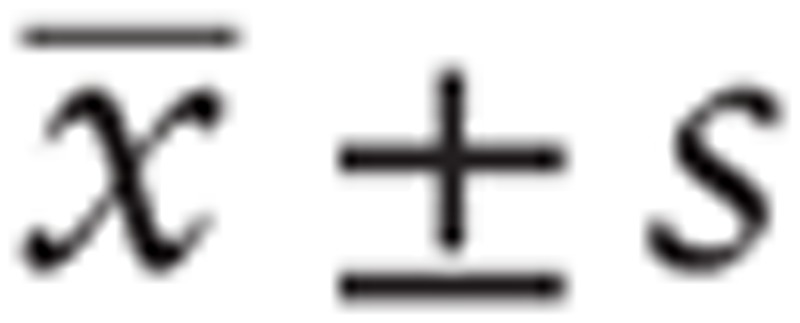
).

**Table 4:**
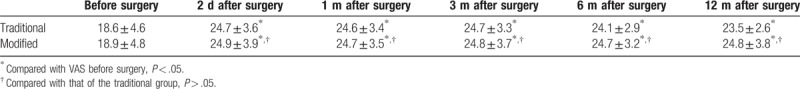
Changes in vertebral height before and after Surgery (
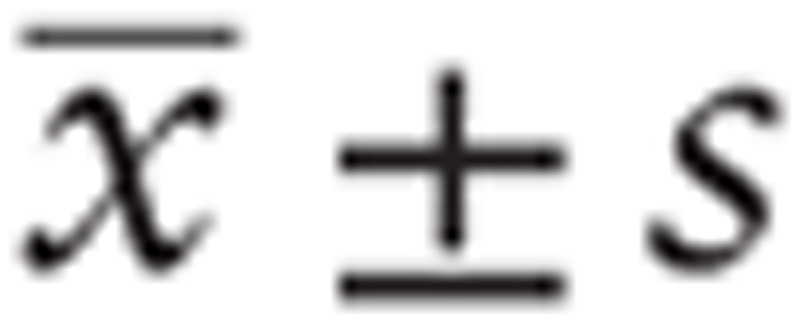
) (mm).

**Table 5:**
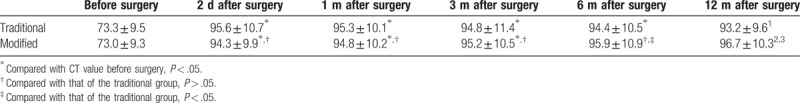
Changes in intra-vertebral CT Value of before and after Surgery (
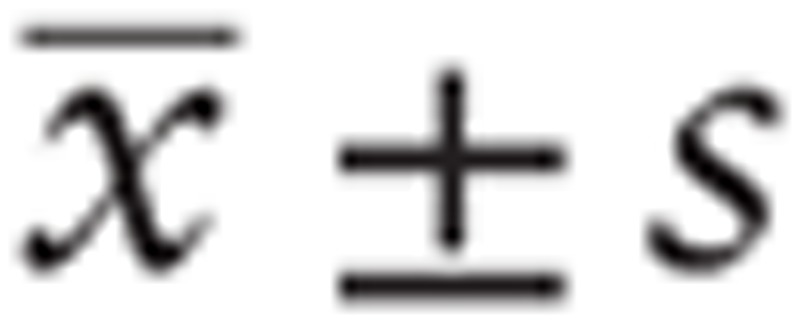
) (HU).

### 3.1 Typical cases

#### 3.1.1 Case 1

Figure 2 shows preoperative and follow-up CT films of a typical case in the modified group. Figure 2A shows the cross section of the fractured vertebra before surgery. Fracture lines and loosely arranged trabeculae of cancellous bone can be identified. Figure 2B shows the cross section of the fractured vertebra 1 month after the surgery with modified bone cement. The modified bone cement is stable within the vertebral body. Good compatibility with adjacent tissue is achieved and there is a noticeable increase in surrounding trabeculae than that before surgery. Figure 2C shows the cross section of the fractured vertebra 6 months after the surgery with modified bone cement. The modified bone cement remains stable within the vertebral body. Good compatibility with adjacent tissue is achieved and there is a noticeable increase in surrounding trabeculae than that before. Figure 2D shows the cross section of the fractured vertebra 1 year after the surgery with modified bone cement. The modified bone cement remains stable within the vertebral body. Further compatibility with adjacent tissue is achieved. The modified bone cement has a tendency to shrink in size. There is a noticeable increase in surrounding trabeculae than that before.

#### 3.1.2 Case 2

**Figure 3:**
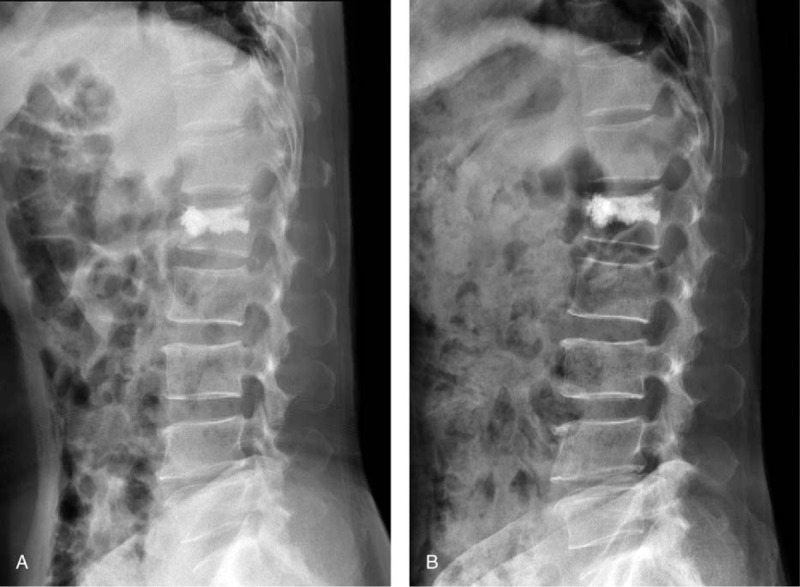
Two days postoperative (A) and 6 months postoperative (B) plain radiographs of a patient in the modified group.

A 70-year-old female patient, whose L1 vertebra fractured due to trauma, was treated by MC modified PMMA bone cement. The modified bone cement was stable and firm within the vertebral body on plain radiograph at the second day after the surgery (Fig. 3A). The patient did not complain pain at her thoracic and lumbar regions 6 months after the surgery. Radiographic follow-up showed the modified PMMA bone cement remained stable and firm within the vertebral body, and there was no wedge-shaped deformation at the adjacent vertebras (Fig. 3B).

## 4 Discussion

The bone cement used in our current OVCF treatment is a mixture mainly of PMMA and solvent, which features injectability, plasticity, and self-coagulability.^[17]^ The constant heat released during coagulation, in vivo, can burn the nerve endings surrounding the fracture to relief pain caused by the compression fracture.^[18,19]^ A stable medium is formed after coagulation and it mechanical strength provides support in the compressed vertebra to restore the height of the fractured vertebral body. Therefore, it is widely used for treatment in the clinical setting. However, disadvantages of PMMA emerge with its use in more patients and the extension of follow-up time,^[20–22]^ including para-vertebral or intra-canal leakage, adjacent vertebral fracture due to high elastic modulus, dislodgment of coagulated bone cement from the vertebral bodies, and other tissue damage due to high coagulation temperature. There is risk of cardio-pulmonary embolism resulting from bone cement leakage before complete coagulation into circulation. Others include non-degradability and poor compatibility with surrounding bone tissue.^[23]^ These have raised increasing concerns among clinicians, especially adjacent vertebral fracture—the main cause of rehospitalization after PVP. According to published literatures in recent years, the incidence of a secondary vertebral fracture after PVP was 12.4% to 27.7% and 66.7% to 76% among which occurred in adjacent levels,^[2,24,25]^ meaning that adjacent vertebral fracture is more likely. Relative reports around the world have revealed a number of influencing factors including type of the bone cement, dosage, leakage, osteoporosis, and distribution of bone cement in the vertebral bodies.^[21,26]^ While the relatively strong stiffness and strength of PMMA bone cement can be used in the treatment, the excessive stiffness of fractured vertebra after surgery transmits mechanical load to adjacent vertebrae and was considered as one of the key reasons lead to adjacent vertebral fracture.^[2,27,28]^

There is an urgent requirement for an ideal bone cement featuring good biocompatibility, biomechanics, osteoinduction, degradability, injectability, and easy clinical manipulation. MC is an artificial biomimetic composite bone graft consisting of orderly arranged type I collagen and hydroxyapatite. The type I collagen was extracted from bovine tendon, and was used as the template for the formation of nano-sized hydroxyapatite through an in vitro biomineralization process.^[29,30]^ The microstructure of MC, possessing similar composition and microstructure of natural bone, accounts for its active osteoinduction and formation of new bone. We mixed certain proportions of MC with different bone cement according to the instructions to prepare MC modified bone cement. The modified bone cement demonstrated good injectability and easy manipulation in the surgery. Postoperative follow-ups revealed lower incidence of leakage and adjacent bone fracture. As adding MC into bone cement during surgery resulted in stiffness reduction and elastic modulus improvement, it is also higher than 70 MPa, meeting the international standards of PMMA bone cement (ISO 5833).^[3]^ Noticeably, the bone density increased more in the 6 months and 1 year follow-ups than that in traditional group, confirming the fact that modified bone cement possessed good osteoinductive activity and biocompatibility. The incidence of postoperative adjacent vertebral fracture dropped to 2% with the modified bone cement, a significant improvement from 13% with the traditional bone cement. The present study has some limitations, such as relative small sample size and short observation time. The present clinical observation is still continued for more and longer clinical outcomes. Multi-center studies with large sample size are needed for further analysis and verification.

In conclusion, with the addition of appropriate proportion of MC, the modified bone cement can achieve identical treatment outcomes to traditional bone cement in pain relief and vertebral height restoration. In the meantime, the modified bone cement features easy clinical manipulation and better clinical outcomes in terms of secondary fracture on adjacent vertebral bodies. Further clinical outcomes of long-term follow-ups are being in progress.

## Author contributions

**Conceptualization:** Jian-Ming Kou, Xi-Sheng Weng, Xi-Feng Zhang.

**Data curation:** Yang Yue.

**Formal analysis:** Jian-Ming Kou, Yang Yue, Zhiye Qiu.

**Investigation:** Xi Wang.

**Methodology:** Xi Wang, Jian-Ming Kou, Xi-Sheng Weng, Zhiye Qiu.

**Project administration:** Jian-Ming Kou.

**Supervision:** Xi-Sheng Weng, Xi-Feng Zhang.

**Writing – original draft:** Xi Wang, Jian-Ming Kou, Yang Yue, Xi-Sheng Weng, Zhiye Qiu, Xi-Feng Zhang.

**Writing – review & editing:** Xi Wang, Jian-Ming Kou, Yang Yue, Xi-Sheng Weng, Zhiye Qiu, Xi-Feng Zhang.
